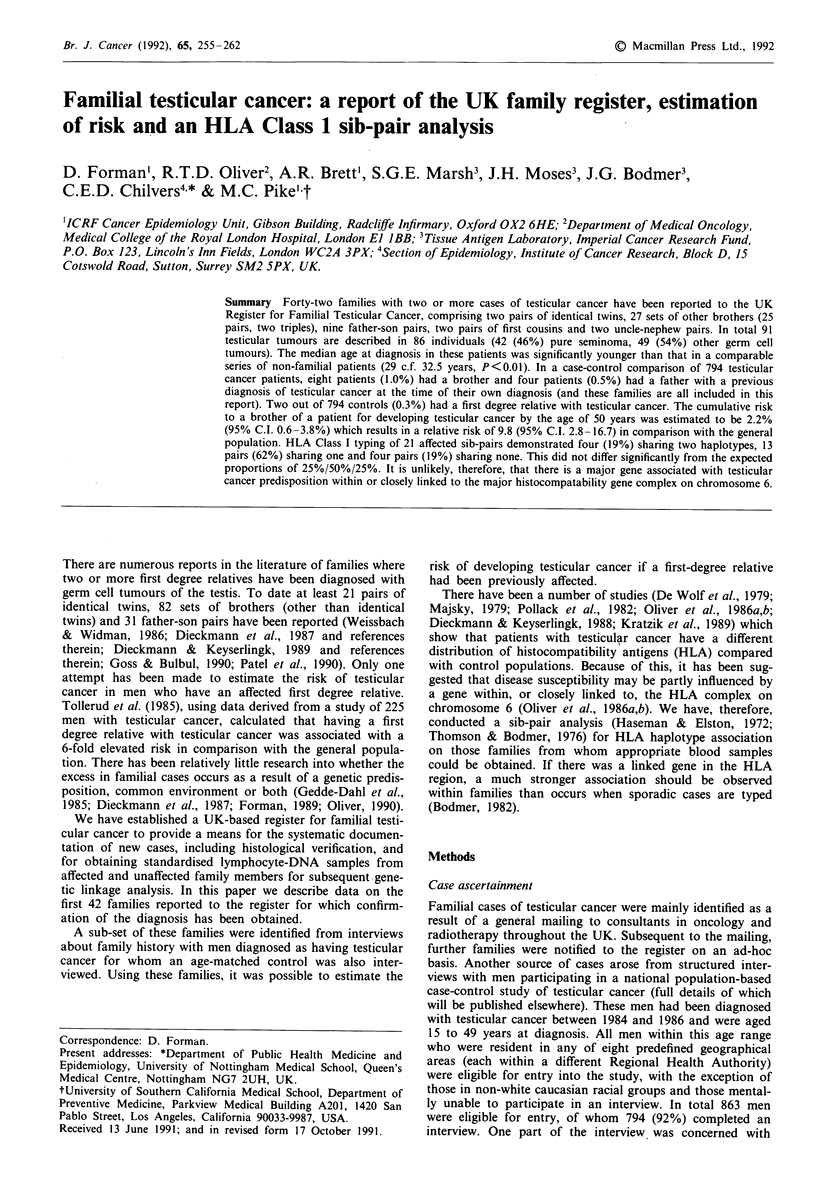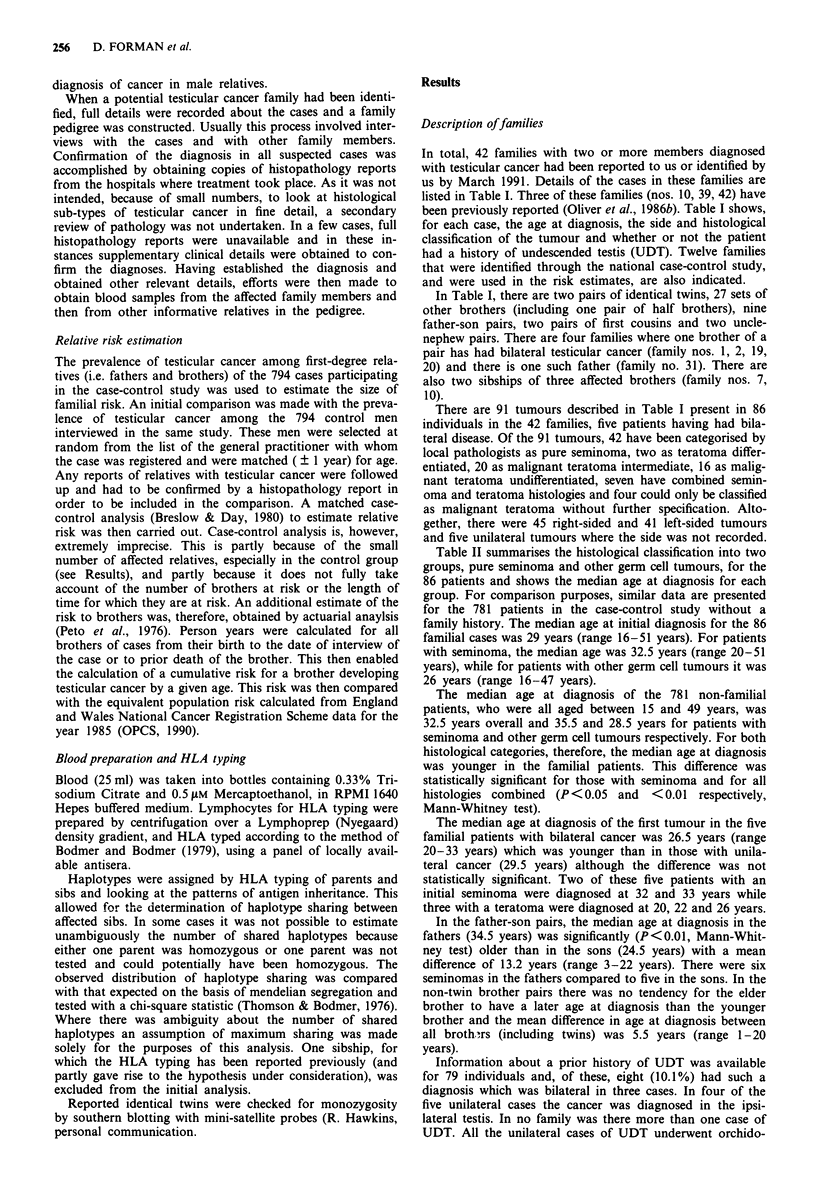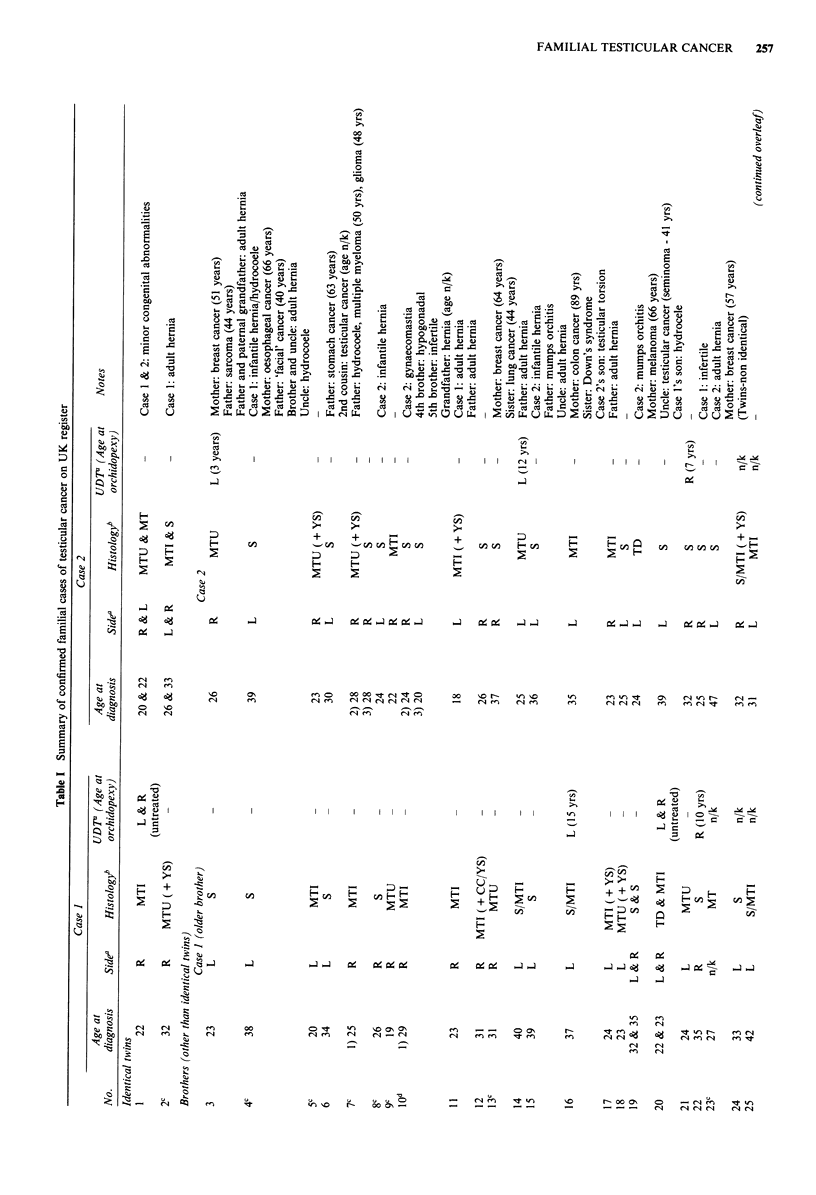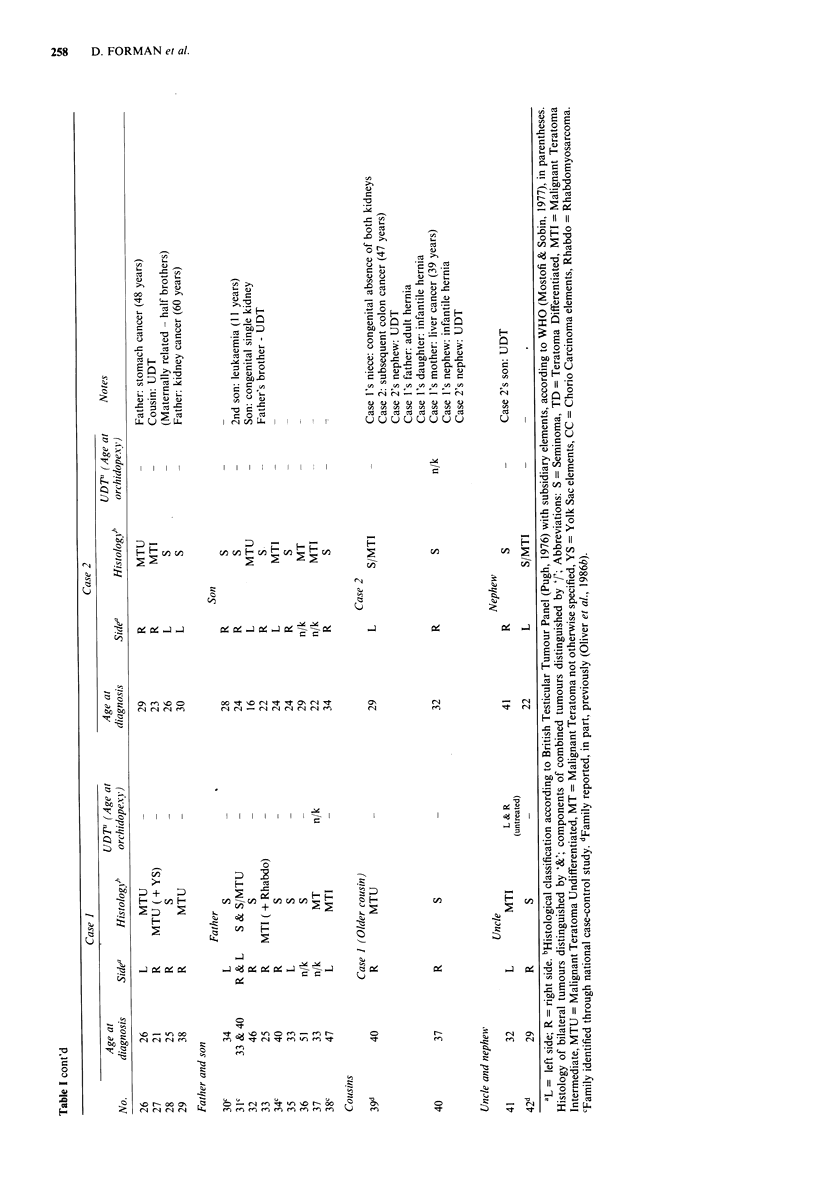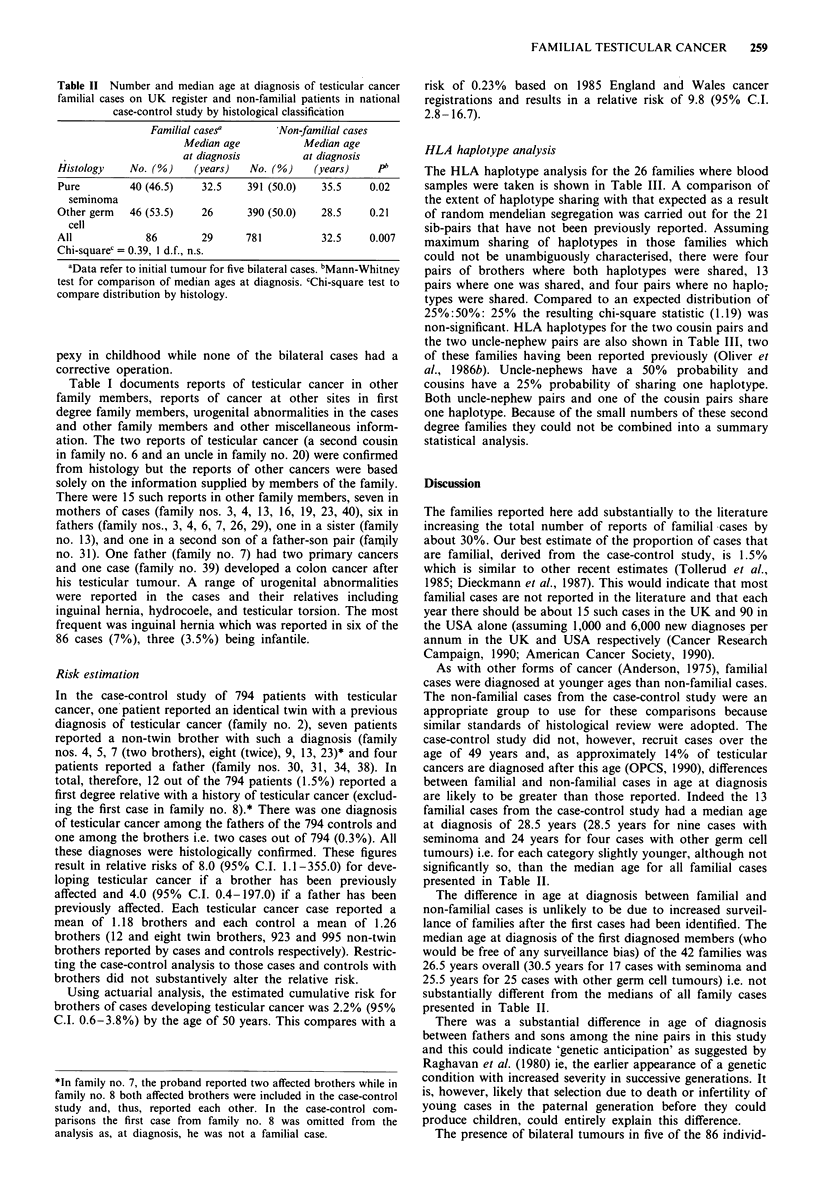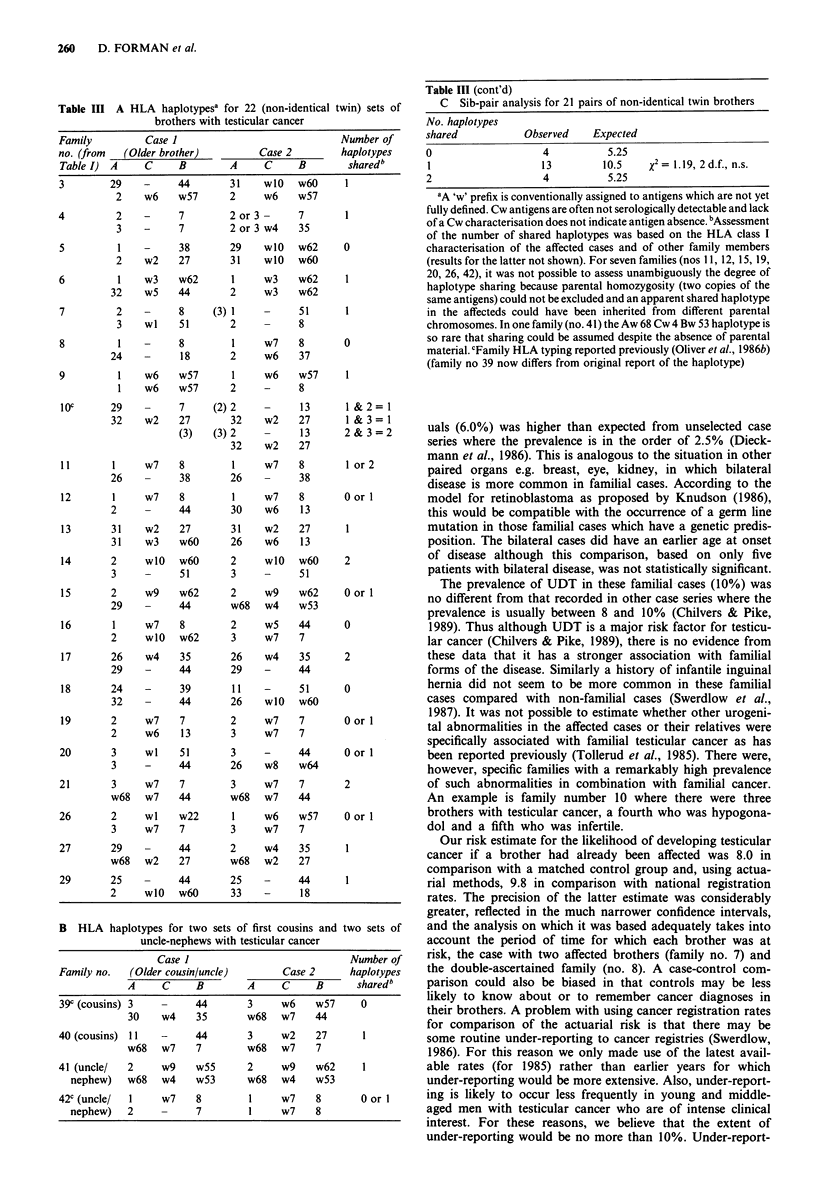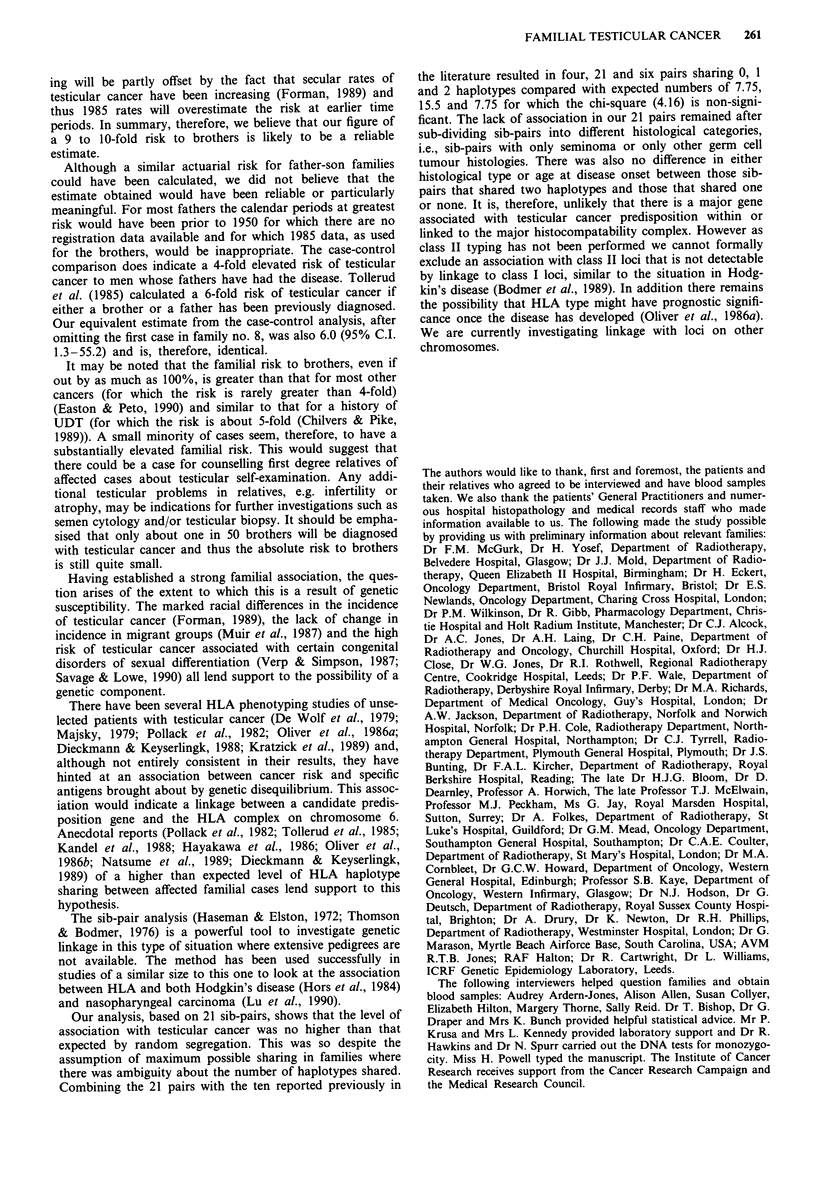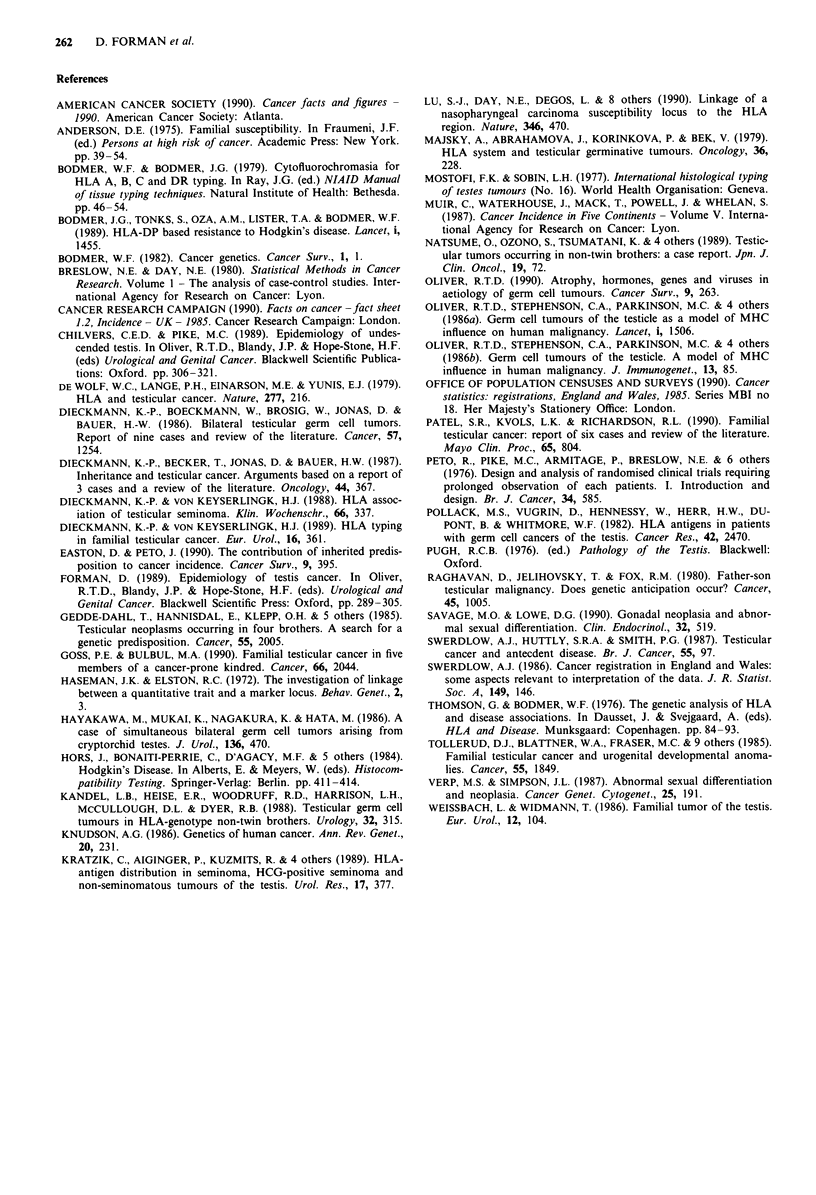# Familial testicular cancer: a report of the UK family register, estimation of risk and an HLA class 1 sib-pair analysis.

**DOI:** 10.1038/bjc.1992.51

**Published:** 1992-02

**Authors:** D. Forman, R. T. Oliver, A. R. Brett, S. G. Marsh, J. H. Moses, J. G. Bodmer, C. E. Chilvers, M. C. Pike

**Affiliations:** ICRF Cancer Epidemiology Unit, Radcliffe Infirmary, Oxford, UK.

## Abstract

Forty-two families with two or more cases of testicular cancer have been reported to the UK Register for Familial Testicular Cancer, comprising two pairs of identical twins, 27 sets of other brothers (25 pairs, two triples), nine father-son pairs, two pairs of first cousins and two uncle-nephew pairs. In total 91 testicular tumours are described in 86 individuals (42 (46%) pure seminoma, 49 (54%) other germ cell tumours). The median age at diagnosis in these patients was significantly younger than that in a comparable series of non-familial patients (29 c.f. 32.5 years, P less than 0.01). In a case-control comparison of 794 testicular cancer patients, eight patients (1.0%) had a brother and four patients (0.5%) had a father with a previous diagnosis of testicular cancer at the time of their own diagnosis (and these families are all included in this report). Two out of 794 controls (0.3%) had a first degree relative with testicular cancer. The cumulative risk to a brother of a patient for developing testicular cancer by the age of 50 years was estimated to be 2.2% (95% C.I. 0.6-3.8%) which results in a relative risk of 9.8 (95% C.I. 2.8-16.7) in comparison with the general population. HLA Class I typing of 21 affected sib-pairs demonstrated four (19%) sharing two haplotypes, 13 pairs (62%) sharing one and four pairs (19%) sharing none. This did not differ significantly from the expected proportions of 25%/50%/25%. It is unlikely, therefore, that there is a major gene associated with testicular cancer predisposition within or closely linked to the major histocompatibility gene complex on chromosome 6.


Br. J. Cancer (1992), 65, 255-262 © Macmillan Press Ltd., 1992

### Familial testicular cancer: a report of the UK family register, estimation of risk and an HLA Class 1 sib-pair analysis

D. Forman[1], R.T.D. Oliver[2], A.R. Brett[1], S.G.E. Marsh[3], J.H. Moses[3], J.G. Bodmer[3], C.E.D. Chilvers[4,*] & M.C. Pike[1,†]

[1]*ICRF Cancer Epidemiology Unit, Gibson Building, Radcliffe Infirmary, Oxford OX2 6HE;* [2]*Department of Medical Oncology, Medical College of the Royal London Hospital, London E1 1BB;* [3]*Tissue Antigen Laboratory, Imperial Cancer Research Fund, P.O. Box 123, Lincoln's Inn Fields, London WC2A 3PX;* [4]*Section of Epidemiology, Institute of Cancer Research, Block D, 15 Cotswold Road, Sutton, Surrey SM2 5PX, UK.*

**Summary** Forty-two families with two or more cases of testicular cancer have been reported to the UK Register for Familial Testicular Cancer, comprising two pairs of identical twins, 27 sets of other brothers (25 pairs, two triples), nine father-son pairs, two pairs of first cousins and two uncle-nephew pairs. In total 91 testicular tumours are described in 86 individuals (42 (46%) pure seminoma, 49 (54%) other germ cell tumours). The median age at diagnosis in these patients was significantly younger than that in a comparable series of non-familial patients (29 c.f. 32.5 years, $P<0.01$). In a case-control comparison of 794 testicular cancer patients, eight patients (1.0%) had a brother and four patients (0.5%) had a father with a previous diagnosis of testicular cancer at the time of their own diagnosis (and these families are all included in this report). Two out of 794 controls (0.3%) had a first degree relative with testicular cancer. The cumulative risk to a brother of a patient for developing testicular cancer by the age of 50 years was estimated to be 2.2% (95% C.I. 0.6-3.8%) which results in a relative risk of 9.8 (95% C.I. 2.8-16.7) in comparison with the general population. HLA Class I typing of 21 affected sib-pairs demonstrated four (19%) sharing two haplotypes, 13 pairs (62%) sharing one and four pairs (19%) sharing none. This did not differ significantly from the expected proportions of 25%/50%/25%. It is unlikely, therefore, that there is a major gene associated with testicular cancer predisposition within or closely linked to the major histocompatability gene complex on chromosome 6.

There are numerous reports in the literature of families where two or more first degree relatives have been diagnosed with germ cell tumours of the testis. To date at least 21 pairs of identical twins, 82 sets of brothers (other than identical twins) and 31 father-son pairs have been reported (Weissbach & Widman, 1986; Dieckmann *et al.*, 1987 and references therein; Dieckmann & Keyserlingk, 1989 and references therein; Goss & Bulbul, 1990; Patel *et al.*, 1990). Only one attempt has been made to estimate the risk of testicular cancer in men who have an affected first degree relative. Tollerud *et al.* (1985), using data derived from a study of 225 men with testicular cancer, calculated that having a first degree relative with testicular cancer was associated with a 6-fold elevated risk in comparison with the general population. There has been relatively little research into whether the excess in familial cases occurs as a result of a genetic predisposition, common environment or both (Gedde-Dahl *et al.*, 1985; Dieckmann *et al.*, 1987; Forman, 1989; Oliver, 1990).

We have established a UK-based register for familial testicular cancer to provide a means for the systematic documentation of new cases, including histological verification, and for obtaining standardised lymphocyte-DNA samples from affected and unaffected family members for subsequent genetic linkage analysis. In this paper we describe data on the first 42 families reported to the register for which confirmation of the diagnosis has been obtained.

A sub-set of these families were identified from interviews about family history with men diagnosed as having testicular cancer for whom an age-matched control was also interviewed. Using these families, it was possible to estimate the risk of developing testicular cancer if a first-degree relative had been previously affected.

There have been a number of studies (De Wolf *et al.*, 1979; Majsky, 1979; Pollack *et al.*, 1982; Oliver *et al.*, 1986*a*,*b*; Dieckmann & Keyserlingk, 1988; Kratzik *et al.*, 1989) which show that patients with testicular cancer have a different distribution of histocompatibility antigens (HLA) compared with control populations. Because of this, it has been suggested that disease susceptibility may be partly influenced by a gene within, or closely linked to, the HLA complex on chromosome 6 (Oliver *et al.*, 1986*a*,*b*). We have, therefore, conducted a sib-pair analysis (Haseman & Elston, 1972; Thomson & Bodmer, 1976) for HLA haplotype association on those families from whom appropriate blood samples could be obtained. If there was a linked gene in the HLA region, a much stronger association should be observed within families than occurs when sporadic cases are typed (Bodmer, 1982).

### Methods

### *Case ascertainment*

Familial cases of testicular cancer were mainly identified as a result of a general mailing to consultants in oncology and radiotherapy throughout the UK. Subsequent to the mailing, further families were notified to the register on an ad-hoc basis. Another source of cases arose from structured interviews with men participating in a national population-based case-control study of testicular cancer (full details of which will be published elsewhere). These men had been diagnosed with testicular cancer between 1984 and 1986 and were aged 15 to 49 years at diagnosis. All men within this age range who were resident in any of eight predefined geographical areas (each within a different Regional Health Authority) were eligible for entry into the study, with the exception of those in non-white caucasian racial groups and those mentally unable to participate in an interview. In total 863 men were eligible for entry, of whom 794 (92%) completed an interview. One part of the interview was concerned with

Correspondence: D. Forman.
Present addresses: *Department of Public Health Medicine and Epidemiology, University of Nottingham Medical School, Queen's Medical Centre, Nottingham NG7 2UH, UK.
†University of Southern California Medical School, Department of Preventive Medicine, Parkview Medical Building A201, 1420 San Pablo Street, Los Angeles, California 90033-9987, USA.
Received 13 June 1991; and in revised form 17 October 1991.

diagnosis of cancer in male relatives.

When a potential testicular cancer family had been identified, full details were recorded about the cases and a family pedigree was constructed. Usually this process involved interviews with the cases and with other family members. Confirmation of the diagnosis in all suspected cases was accomplished by obtaining copies of histopathology reports from the hospitals where treatment took place. As it was not intended, because of small numbers, to look at histological sub-types of testicular cancer in fine detail, a secondary review of pathology was not undertaken. In a few cases, full histopathology reports were unavailable and in these instances supplementary clinical details were obtained to confirm the diagnoses. Having established the diagnosis and obtained other relevant details, efforts were then made to obtain blood samples from the affected family members and then from other informative relatives in the pedigree.

### *Relative risk estimation*

The prevalence of testicular cancer among first-degree relatives (i.e. fathers and brothers) of the 794 cases participating in the case-control study was used to estimate the size of familial risk. An initial comparison was made with the prevalence of testicular cancer among the 794 control men interviewed in the same study. These men were selected at random from the list of the general practitioner with whom the case was registered and were matched ($\pm$ 1 year) for age. Any reports of relatives with testicular cancer were followed up and had to be confirmed by a histopathology report in order to be included in the comparison. A matched case-control analysis (Breslow & Day, 1980) to estimate relative risk was then carried out. Case-control analysis is, however, extremely imprecise. This is partly because of the small number of affected relatives, especially in the control group (see Results), and partly because it does not fully take account of the number of brothers at risk or the length of time for which they are at risk. An additional estimate of the risk to brothers was, therefore, obtained by actuarial anaylsis (Peto *et al.*, 1976). Person years were calculated for all brothers of cases from their birth to the date of interview of the case or to prior death of the brother. This then enabled the calculation of a cumulative risk for a brother developing testicular cancer by a given age. This risk was then compared with the equivalent population risk calculated from England and Wales National Cancer Registration Scheme data for the year 1985 (OPCS, 1990).

### *Blood preparation and HLA typing*

Blood (25 ml) was taken into bottles containing 0.33% Trisodium Citrate and 0.5 $\mu$M Mercaptoethanol, in RPMI 1640 Hepes buffered medium. Lymphocytes for HLA typing were prepared by centrifugation over a Lymphoprep (Nyegaard) density gradient, and HLA typed according to the method of Bodmer and Bodmer (1979), using a panel of locally available antisera.

Haplotypes were assigned by HLA typing of parents and sibs and looking at the patterns of antigen inheritance. This allowed for the determination of haplotype sharing between affected sibs. In some cases it was not possible to estimate unambiguously the number of shared haplotypes because either one parent was homozygous or one parent was not tested and could potentially have been homozygous. The observed distribution of haplotype sharing was compared with that expected on the basis of mendelian segregation and tested with a chi-square statistic (Thomson & Bodmer, 1976). Where there was ambiguity about the number of shared haplotypes an assumption of maximum sharing was made solely for the purposes of this analysis. One sibship, for which the HLA typing has been reported previously (and partly gave rise to the hypothesis under consideration), was excluded from the initial analysis.

Reported identical twins were checked for monozygosity by southern blotting with mini-satellite probes (R. Hawkins, personal communication.

### Results

### *Description of families*

In total, 42 families with two or more members diagnosed with testicular cancer had been reported to us or identified by us by March 1991. Details of the cases in these families are listed in Table I. Three of these families (nos. 10, 39, 42) have been previously reported (Oliver *et al.*, 1986*b*). Table I shows, for each case, the age at diagnosis, the side and histological classification of the tumour and whether or not the patient had a history of undescended testis (UDT). Twelve families that were identified through the national case-control study, and were used in the risk estimates, are also indicated.

In Table I, there are two pairs of identical twins, 27 sets of other brothers (including one pair of half brothers), nine father-son pairs, two pairs of first cousins and two uncle-nephew pairs. There are four families where one brother of a pair has had bilateral testicular cancer (family nos. 1, 2, 19, 20) and there is one such father (family no. 31). There are also two sibships of three affected brothers (family nos. 7, 10).

There are 91 tumours described in Table I present in 86 individuals in the 42 families, five patients having had bilateral disease. Of the 91 tumours, 42 have been categorised by local pathologists as pure seminoma, two as teratoma differentiated, 20 as malignant teratoma intermediate, 16 as malignant teratoma undifferentiated, seven have combined seminoma and teratoma histologies and four could only be classified as malignant teratoma without further specification. Altogether, there were 45 right-sided and 41 left-sided tumours and five unilateral tumours where the side was not recorded.

Table II summarises the histological classification into two groups, pure seminoma and other germ cell tumours, for the 86 patients and shows the median age at diagnosis for each group. For comparison purposes, similar data are presented for the 781 patients in the case-control study without a family history. The median age at initial diagnosis for the 86 familial cases was 29 years (range 16–51 years). For patients with seminoma, the median age was 32.5 years (range 20–51 years), while for patients with other germ cell tumours it was 26 years (range 16–47 years).

The median age at diagnosis of the 781 non-familial patients, who were all aged between 15 and 49 years, was 32.5 years overall and 35.5 and 28.5 years for patients with seminoma and other germ cell tumours respectively. For both histological categories, therefore, the median age at diagnosis was younger in the familial patients. This difference was statistically significant for those with seminoma and for all histologies combined ($P<0.05$ and $<0.01$ respectively, Mann-Whitney test).

The median age at diagnosis of the first tumour in the five familial patients with bilateral cancer was 26.5 years (range 20–33 years) which was younger than in those with unilateral cancer (29.5 years) although the difference was not statistically significant. Two of these five patients with an initial seminoma were diagnosed at 32 and 33 years while three with a teratoma were diagnosed at 20, 22 and 26 years.

In the father-son pairs, the median age at diagnosis in the fathers (34.5 years) was significantly ($P<0.01$, Mann-Whitney test) older than in the sons (24.5 years) with a mean difference of 13.2 years (range 3–22 years). There were six seminomas in the fathers compared to five in the sons. In the non-twin brother pairs there was no tendency for the elder brother to have a later age at diagnosis than the younger brother and the mean difference in age at diagnosis between all brothers (including twins) was 5.5 years (range 1–20 years).

Information about a prior history of UDT was available for 79 individuals and, of these, eight (10.1%) had such a diagnosis which was bilateral in three cases. In four of the five unilateral cases the cancer was diagnosed in the ipsilateral testis. In no family was there more than one case of UDT. All the unilateral cases of UDT underwent orchido-

**Table I** Summary of confirmed familial cases of testicular cancer on UK register

| | Case 1 | | | | Case 2 | | | | |
|---|---|---|---|---|---|---|---|---|---|
| No. | Age at diagnosis | Side[a] | Histology[b] | UDT[a] (Age at orchidopexy) | Age at diagnosis | Side[a] | Histology[b] | UDT[a] (Age at orchidopexy) | Notes |
| *Identical twins* | | | | | | | | | |
| 1 | 22 | R | MTI | L & R (untreated) | 20 & 22 | R & L | MTU & MT | – | Case 1 & 2: minor congenital abnormalities |
| 2[c] | 32 | R | MTU ( + YS) | – | 26 & 33 | L & R | MTI & S | – | Case 1: adult hernia |
| *Brothers (other than identical twins)* | | | | | | | | | |
| | *Case 1 (older brother)* | | | | *Case 2* | | | | |
| 3 | 23 | L | S | – | 26 | R | MTU | L (3 years) | Mother: breast cancer (51 years)<br>Father: sarcoma (44 years)<br>Father and paternal grandfather: adult hernia |
| 4[c] | 38 | L | S | – | 39 | L | S | – | Case 1: infantile hernia/hydrocoele<br>Mother: oesophageal cancer (66 years)<br>Father: 'facial' cancer (40 years)<br>Brother and uncle: adult hernia<br>Uncle: hydrocoele |
| 5[c] | 20 | L | MTI | – | 23 | R | MTU ( + YS) | – | – |
| 6 | 34 | L | S | – | 30 | L | S | – | Father: stomach cancer (63 years)<br>2nd cousin: testicular cancer (age n/k) |
| 7[c] | 1) 25 | R | MTI | – | 2) 28 | R | MTU (+ YS) | – | Father: hydrocoele, multiple myeloma (50 yrs), glioma (48 yrs) |
| | | | | | 3) 28 | R | S | – | |
| 8[c] | 26 | R | S | – | 24 | L | S | – | Case 2: infantile hernia |
| 9[c] | 19 | R | MTU | – | 22 | R | MTI | – | – |
| 10[d] | 1) 29 | R | MTI | – | 2) 24 | R | S | – | Case 2: gynaecomastia |
| | | | | | 3) 20 | L | S | | 4th brother: hypogonadal<br>5th brother: infertile<br>Grandfather: hernia (age n/k) |
| 11 | 23 | R | MTI | – | 18 | L | MTI ( + YS) | – | Case 1: adult hernia<br>Father: adult hernia |
| 12 | 31 | R | MTI ( + CC/YS) | – | 26 | R | S | – | – |
| 13[c] | 31 | R | MTU | – | 37 | R | S | – | Mother: breast cancer (64 years)<br>Sister: lung cancer (44 years) |
| 14 | 40 | L | S/MTI | – | 25 | L | MTU | L (12 yrs) | Father: adult hernia |
| 15 | 39 | L | S | – | 36 | L | S | – | Case 2: infantile hernia<br>Father: mumps orchitis<br>Uncle: adult hernia |
| 16 | 37 | L | S/MTI | L (15 yrs) | 35 | L | MTI | – | Mother: colon cancer (89 yrs)<br>Sister: Down's syndrome<br>Case 2's son: testicular torsion |
| 17 | 24 | L | MTI ( + YS) | – | 23 | R | MTI | – | Father: adult hernia |
| 18 | 23 | L | MTU ( + YS) | – | 25 | L | S | – | – |
| 19 | 32 & 35 | L & R | S & S | – | 24 | L | TD | – | Case 2: mumps orchitis<br>Mother: melanoma (66 years) |
| 20 | 22 & 23 | L & R | TD & MTI | L & R (untreated) | 39 | L | S | – | Uncle: testicular cancer (seminoma - 41 yrs)<br>Case 1's son: hydrocele |
| 21 | 24 | L | MTU | – | 32 | R | S | R (7 yrs) | – |
| 22 | 35 | R | S | R (10 yrs) | 25 | R | S | – | Case 1: infertile |
| 23[c] | 27 | n/k | MT | n/k | 47 | L | S | – | Case 2: adult hernia<br>Mother: breast cancer (57 years) |
| 24 | 33 | L | S | n/k | 32 | R | S/MTI ( + YS) | n/k | (Twins-non identical) |
| 25 | 42 | L | S/MTI | n/k | 31 | L | MTI | n/k | – |

*(continued overleaf)*

**Table I** cont'd

| No. | Case 1: Age at diagnosis | Case 1: Side[a] | Case 1: Histology[b] | Case 1: UDT[a] (Age at orchidopexy) | Case 2: Age at diagnosis | Case 2: Side[a] | Case 2: Histology[b] | Case 2: UDT[a] (Age at orchidopexy) | Notes |
|---|---|---|---|---|---|---|---|---|---|
| 26 | 26 | L | MTU | – | 29 | R | MTU | – | Father: stomach cancer (48 years) |
| 27 | 21 | R | MTU ( + YS) | – | 23 | R | MTI | – | Cousin: UDT |
| 28 | 25 | R | S | – | 26 | L | S | – | (Maternally related – half brothers) |
| 29 | 38 | R | MTU | – | 30 | L | S | – | Father: kidney cancer (60 years) |
| *Father and son* | | | | | | | | | |
| | | | *Father* | | | | *Son* | | |
| 30[c] | 34 | L | S | – | 28 | R | S | – | – |
| 31[c] | 33 & 40 | R & L | S & S/MTU | – | 24 | R | S | – | 2nd son: leukaemia (11 years) |
| 32 | 46 | R | S | – | 16 | L | MTU | – | Son: congenital single kidney |
| 33 | 25 | R | MTI ( + Rhabdo) | – | 22 | R | S | – | Father's brother - UDT |
| 34[c] | 40 | R | S | – | 24 | L | MTI | – | – |
| 35 | 33 | L | S | – | 24 | R | S | – | – |
| 36 | 51 | n/k | S | – | 29 | n/k | MT | – | – |
| 37 | 33 | n/k | MT | n/k | 22 | n/k | MTI | – | – |
| 38[c] | 47 | L | MTI | – | 34 | R | S | – | – |
| *Cousins* | | | | | | | | | |
| | | | *Case 1 (Older cousin)* | | | | *Case 2* | | |
| 39[d] | 40 | R | MTU | – | 29 | L | S/MTI | – | Case 1's niece: congenital absence of both kidneys<br>Case 2: subsequent colon cancer (47 years)<br>Case 2's nephew: UDT |
| 40 | 37 | R | S | – | 32 | R | S | n/k | Case 1's father: adult hernia<br>Case 1's daughter: infantile hernia<br>Case 1's mother: liver cancer (39 years)<br>Case 1's nephew: infantile hernia<br>Case 2's nephew: UDT |
| *Uncle and nephew* | | | | | | | | | |
| | | | *Uncle* | | | | *Nephew* | | |
| 41 | 32 | L | MTI | L & R (untreated) | 41 | R | S | – | Case 2's son: UDT |
| 42[d] | 29 | R | S | – | 22 | L | S/MTI | – | – |

[a]L = left side; R = right side. [b]Histological classification according to British Testicular Tumour Panel (Pugh, 1976) with subsidiary elements, according to WHO (Mostofi & Sobin, 1977), in parentheses. Histology of bilateral tumours distinguished by '&'; components of combined tumours distinguished by '/'; Abbreviations: S = Seminoma, TD = Teratoma Differentiated, MTI = Malignant Teratoma Intermediate, MTU = Malignant Teratoma Undifferentiated, MT = Malignant Teratoma not otherwise specified, YS = Yolk Sac elements, CC = Chorio Carcinoma elements, Rhabdo = Rhabdomyosarcoma. [c]Family identified through national case-control study. [d]Family reported, in part, previously (Oliver *et al.*, 1986*b*).

**Table II** Number and median age at diagnosis of testicular cancer familial cases on UK register and non-familial patients in national case-control study by histological classification

| | Familial cases[a] | | Non-familial cases | | |
|---|---|---|---|---|---|
| *Histology* | *No. (%)* | *Median age at diagnosis (years)* | *No. (%)* | *Median age at diagnosis (years)* | $P^b$ |
| Pure seminoma | 40 (46.5) | 32.5 | 391 (50.0) | 35.5 | 0.02 |
| Other germ cell | 46 (53.5) | 26 | 390 (50.0) | 28.5 | 0.21 |
| All | 86 | 29 | 781 | 32.5 | 0.007 |
| Chi-square[c] = 0.39, 1 d.f., n.s. | | | | | |

[a]Data refer to initial tumour for five bilateral cases. [b]Mann-Whitney test for comparison of median ages at diagnosis. [c]Chi-square test to compare distribution by histology.

pexy in childhood while none of the bilateral cases had a corrective operation.

Table I documents reports of testicular cancer in other family members, reports of cancer at other sites in first degree family members, urogenital abnormalities in the cases and other family members and other miscellaneous information. The two reports of testicular cancer (a second cousin in family no. 6 and an uncle in family no. 20) were confirmed from histology but the reports of other cancers were based solely on the information supplied by members of the family. There were 15 such reports in other family members, seven in mothers of cases (family nos. 3, 4, 13, 16, 19, 23, 40), six in fathers (family nos., 3, 4, 6, 7, 26, 29), one in a sister (family no. 13), and one in a second son of a father-son pair (family no. 31). One father (family no. 7) had two primary cancers and one case (family no. 39) developed a colon cancer after his testicular tumour. A range of urogenital abnormalities were reported in the cases and their relatives including inguinal hernia, hydrocoele, and testicular torsion. The most frequent was inguinal hernia which was reported in six of the 86 cases (7%), three (3.5%) being infantile.

### *Risk estimation*

In the case-control study of 794 patients with testicular cancer, one patient reported an identical twin with a previous diagnosis of testicular cancer (family no. 2), seven patients reported a non-twin brother with such a diagnosis (family nos. 4, 5, 7 (two brothers), eight (twice), 9, 13, 23)* and four patients reported a father (family nos. 30, 31, 34, 38). In total, therefore, 12 out of the 794 patients (1.5%) reported a first degree relative with a history of testicular cancer (excluding the first case in family no. 8).* There was one diagnosis of testicular cancer among the fathers of the 794 controls and one among the brothers i.e. two cases out of 794 (0.3%). All these diagnoses were histologically confirmed. These figures result in relative risks of 8.0 (95% C.I. 1.1–355.0) for developing testicular cancer if a brother has been previously affected and 4.0 (95% C.I. 0.4–197.0) if a father has been previously affected. Each testicular cancer case reported a mean of 1.18 brothers and each control a mean of 1.26 brothers (12 and eight twin brothers, 923 and 995 non-twin brothers reported by cases and controls respectively). Restricting the case-control analysis to those cases and controls with brothers did not substantively alter the relative risk.

Using actuarial analysis, the estimated cumulative risk for brothers of cases developing testicular cancer was 2.2% (95% C.I. 0.6–3.8%) by the age of 50 years. This compares with a risk of 0.23% based on 1985 England and Wales cancer registrations and results in a relative risk of 9.8 (95% C.I. 2.8–16.7).

*In family no. 7, the proband reported two affected brothers while in family no. 8 both affected brothers were included in the case-control study and, thus, reported each other. In the case-control comparisons the first case from family no. 8 was omitted from the analysis as, at diagnosis, he was not a familial case.

### *HLA haplotype analysis*

The HLA haplotype analysis for the 26 families where blood samples were taken is shown in Table III. A comparison of the extent of haplotype sharing with that expected as a result of random mendelian segregation was carried out for the 21 sib-pairs that have not been previously reported. Assuming maximum sharing of haplotypes in those families which could not be unambiguously characterised, there were four pairs of brothers where both haplotypes were shared, 13 pairs where one was shared, and four pairs where no haplotypes were shared. Compared to an expected distribution of 25%:50%: 25% the resulting chi-square statistic (1.19) was non-significant. HLA haplotypes for the two cousin pairs and the two uncle-nephew pairs are also shown in Table III, two of these families having been reported previously (Oliver *et al.*, 1986*b*). Uncle-nephews have a 50% probability and cousins have a 25% probability of sharing one haplotype. Both uncle-nephew pairs and one of the cousin pairs share one haplotype. Because of the small numbers of these second degree families they could not be combined into a summary statistical analysis.

### Discussion

The families reported here add substantially to the literature increasing the total number of reports of familial cases by about 30%. Our best estimate of the proportion of cases that are familial, derived from the case-control study, is 1.5% which is similar to other recent estimates (Tollerud *et al.*, 1985; Dieckmann *et al.*, 1987). This would indicate that most familial cases are not reported in the literature and that each year there should be about 15 such cases in the UK and 90 in the USA alone (assuming 1,000 and 6,000 new diagnoses per annum in the UK and USA respectively (Cancer Research Campaign, 1990; American Cancer Society, 1990).

As with other forms of cancer (Anderson, 1975), familial cases were diagnosed at younger ages than non-familial cases. The non-familial cases from the case-control study were an appropriate group to use for these comparisons because similar standards of histological review were adopted. The case-control study did not, however, recruit cases over the age of 49 years and, as approximately 14% of testicular cancers are diagnosed after this age (OPCS, 1990), differences between familial and non-familial cases in age at diagnosis are likely to be greater than those reported. Indeed the 13 familial cases from the case-control study had a median age at diagnosis of 28.5 years (28.5 years for nine cases with seminoma and 24 years for four cases with other germ cell tumours) i.e. for each category slightly younger, although not significantly so, than the median age for all familial cases presented in Table II.

The difference in age at diagnosis between familial and non-familial cases is unlikely to be due to increased surveillance of families after the first cases had been identified. The median age at diagnosis of the first diagnosed members (who would be free of any surveillance bias) of the 42 families was 26.5 years overall (30.5 years for 17 cases with seminoma and 25.5 years for 25 cases with other germ cell tumours) i.e. not substantially different from the medians of all family cases presented in Table II.

There was a substantial difference in age of diagnosis between fathers and sons among the nine pairs in this study and this could indicate 'genetic anticipation' as suggested by Raghavan *et al.* (1980) ie, the earlier appearance of a genetic condition with increased severity in successive generations. It is, however, likely that selection due to death or infertility of young cases in the paternal generation before they could produce children, could entirely explain this difference.

The presence of bilateral tumours in five of the 86 individ-

**Table III** A HLA haplotypes[a] for 22 (non-identical twin) sets of brothers with testicular cancer

| Family no. (from Table I) | Case 1 (Older brother) A | C | B | Case 2 A | C | B | Number of haplotypes shared[b] |
|---|---|---|---|---|---|---|---|
| 3 | 29 | – | 44 | 31 | w10 | w60 | 1 |
| | 2 | w6 | w57 | 2 | w6 | w57 | |
| 4 | 2 | – | 7 | 2 or 3 | – | 7 | 1 |
| | 3 | – | 7 | 2 or 3 | w4 | 35 | |
| 5 | 1 | – | 38 | 29 | w10 | w62 | 0 |
| | 2 | w2 | 27 | 31 | w10 | w60 | |
| 6 | 1 | w3 | w62 | 1 | w3 | w62 | 1 |
| | 32 | w5 | 44 | 2 | w3 | w62 | |
| 7 | 2 | – | 8 | (3) 1 | – | 51 | 1 |
| | 3 | w1 | 51 | 2 | – | 8 | |
| 8 | 1 | – | 8 | 1 | w7 | 8 | 0 |
| | 24 | – | 18 | 2 | w6 | 37 | |
| 9 | 1 | w6 | w57 | 1 | w6 | w57 | 1 |
| | 1 | w6 | w57 | 2 | – | 8 | |
| 10[c] | 29 | – | 7 | (2) 2 | – | 13 | 1 & 2 = 1 |
| | 32 | w2 | 27 | 32 | w2 | 27 | 1 & 3 = 1 |
| | | | (3) | (3) 2 | – | 13 | 2 & 3 = 2 |
| | | | | 32 | w2 | 27 | |
| 11 | 1 | w7 | 8 | 1 | w7 | 8 | 1 or 2 |
| | 26 | – | 38 | 26 | – | 38 | |
| 12 | 1 | w7 | 8 | 1 | w7 | 8 | 0 or 1 |
| | 2 | – | 44 | 30 | w6 | 13 | |
| 13 | 31 | w2 | 27 | 31 | w2 | 27 | 1 |
| | 31 | w3 | w60 | 26 | w6 | 13 | |
| 14 | 2 | w10 | w60 | 2 | w10 | w60 | 2 |
| | 3 | – | 51 | 3 | – | 51 | |
| 15 | 2 | w9 | w62 | 2 | w9 | w62 | 0 or 1 |
| | 29 | – | 44 | w68 | w4 | w53 | |
| 16 | 1 | w7 | 8 | 2 | w5 | 44 | 0 |
| | 2 | w10 | w62 | 3 | w7 | 7 | |
| 17 | 26 | w4 | 35 | 26 | w4 | 35 | 2 |
| | 29 | – | 44 | 29 | – | 44 | |
| 18 | 24 | – | 39 | 11 | – | 51 | 0 |
| | 32 | – | 44 | 26 | w10 | w60 | |
| 19 | 2 | w7 | 7 | 2 | w7 | 7 | 0 or 1 |
| | 2 | w6 | 13 | 3 | w7 | 7 | |
| 20 | 3 | w1 | 51 | 3 | – | 44 | 0 or 1 |
| | 3 | – | 44 | 26 | w8 | w64 | |
| 21 | 3 | w7 | 7 | 3 | w7 | 7 | 2 |
| | w68 | w7 | 44 | w68 | w7 | 44 | |
| 26 | 2 | w1 | w22 | 1 | w6 | w57 | 0 or 1 |
| | 3 | w7 | 7 | 3 | w7 | 7 | |
| 27 | 29 | – | 44 | 2 | w4 | 35 | 1 |
| | w68 | w2 | 27 | w68 | w2 | 27 | |
| 29 | 25 | – | 44 | 25 | – | 44 | 1 |
| | 2 | w10 | w60 | 33 | – | 18 | |

**B** HLA haplotypes for two sets of first cousins and two sets of uncle-nephews with testicular cancer

| Family no. | Case 1 (Older cousin/uncle) A | C | B | Case 2 A | C | B | Number of haplotypes shared[b] |
|---|---|---|---|---|---|---|---|
| 39[c] (cousins) | 3 | – | 44 | 3 | w6 | w57 | 0 |
| | 30 | w4 | 35 | w68 | w7 | 44 | |
| 40 (cousins) | 11 | – | 44 | 3 | w2 | 27 | 1 |
| | w68 | w7 | 7 | w68 | w7 | 7 | |
| 41 (uncle/ nephew) | 2 | w9 | w55 | 2 | w9 | w62 | 1 |
| | w68 | w4 | w53 | w68 | w4 | w53 | |
| 42[c] (uncle/ nephew) | 1 | w7 | 8 | 1 | w7 | 8 | 0 or 1 |
| | 2 | – | 7 | 1 | w7 | 8 | |

**Table III** (cont'd)
C Sib-pair analysis for 21 pairs of non-identical twin brothers

| No. haplotypes shared | Observed | Expected | |
|---|---|---|---|
| 0 | 4 | 5.25 | |
| 1 | 13 | 10.5 | $\chi^2 = 1.19$, 2 d.f., n.s. |
| 2 | 4 | 5.25 | |

[a]A 'w' prefix is conventionally assigned to antigens which are not yet fully defined. Cw antigens are often not serologically detectable and lack of a Cw characterisation does not indicate antigen absence. [b]Assessment of the number of shared haplotypes was based on the HLA class I characterisation of the affected cases and of other family members (results for the latter not shown). For seven families (nos 11, 12, 15, 19, 20, 26, 42), it was not possible to assess unambiguously the degree of haplotype sharing because parental homozygosity (two copies of the same antigens) could not be excluded and an apparent shared haplotype in the affecteds could have been inherited from different parental chromosomes. In one family (no. 41) the Aw 68 Cw 4 Bw 53 haplotype is so rare that sharing could be assumed despite the absence of parental material. [c]Family HLA typing reported previously (Oliver et al., 1986b) (family no 39 now differs from original report of the haplotype)

uals (6.0%) was higher than expected from unselected case series where the prevalence is in the order of 2.5% (Dieckmann et al., 1986). This is analogous to the situation in other paired organs e.g. breast, eye, kidney, in which bilateral disease is more common in familial cases. According to the model for retinoblastoma as proposed by Knudson (1986), this would be compatible with the occurrence of a germ line mutation in those familial cases which have a genetic predisposition. The bilateral cases did have an earlier age at onset of disease although this comparison, based on only five patients with bilateral disease, was not statistically significant.

The prevalence of UDT in these familial cases (10%) was no different from that recorded in other case series where the prevalence is usually between 8 and 10% (Chilvers & Pike, 1989). Thus although UDT is a major risk factor for testicular cancer (Chilvers & Pike, 1989), there is no evidence from these data that it has a stronger association with familial forms of the disease. Similarly a history of infantile inguinal hernia did not seem to be more common in these familial cases compared with non-familial cases (Swerdlow et al., 1987). It was not possible to estimate whether other urogenital abnormalities in the affected cases or their relatives were specifically associated with familial testicular cancer as has been reported previously (Tollerud et al., 1985). There were, however, specific families with a remarkably high prevalence of such abnormalities in combination with familial cancer. An example is family number 10 where there were three brothers with testicular cancer, a fourth who was hypogonadol and a fifth who was infertile.

Our risk estimate for the likelihood of developing testicular cancer if a brother had already been affected was 8.0 in comparison with a matched control group and, using actuarial methods, 9.8 in comparison with national registration rates. The precision of the latter estimate was considerably greater, reflected in the much narrower confidence intervals, and the analysis on which it was based adequately takes into account the period of time for which each brother was at risk, the case with two affected brothers (family no. 7) and the double-ascertained family (no. 8). A case-control comparison could also be biased in that controls may be less likely to know about or to remember cancer diagnoses in their brothers. A problem with using cancer registration rates for comparison of the actuarial risk is that there may be some routine under-reporting to cancer registries (Swerdlow, 1986). For this reason we only made use of the latest available rates (for 1985) rather than earlier years for which under-reporting would be more extensive. Also, under-reporting is likely to occur less frequently in young and middle-aged men with testicular cancer who are of intense clinical interest. For these reasons, we believe that the extent of under-reporting would be no more than 10%. Under-reporting will be partly offset by the fact that secular rates of testicular cancer have been increasing (Forman, 1989) and thus 1985 rates will overestimate the risk at earlier time periods. In summary, therefore, we believe that our figure of a 9 to 10-fold risk to brothers is likely to be a reliable estimate.

Although a similar actuarial risk for father-son families could have been calculated, we did not believe that the estimate obtained would have been reliable or particularly meaningful. For most fathers the calendar periods at greatest risk would have been prior to 1950 for which there are no registration data available and for which 1985 data, as used for the brothers, would be inappropriate. The case-control comparison does indicate a 4-fold elevated risk of testicular cancer to men whose fathers have had the disease. Tollerud *et al.* (1985) calculated a 6-fold risk of testicular cancer if either a brother or a father has been previously diagnosed. Our equivalent estimate from the case-control analysis, after omitting the first case in family no. 8, was also 6.0 (95% C.I. 1.3–55.2) and is, therefore, identical.

It may be noted that the familial risk to brothers, even if out by as much as 100%, is greater than that for most other cancers (for which the risk is rarely greater than 4-fold) (Easton & Peto, 1990) and similar to that for a history of UDT (for which the risk is about 5-fold (Chilvers & Pike, 1989)). A small minority of cases seem, therefore, to have a substantially elevated familial risk. This would suggest that there could be a case for counselling first degree relatives of affected cases about testicular self-examination. Any additional testicular problems in relatives, e.g. infertility or atrophy, may be indications for further investigations such as semen cytology and/or testicular biopsy. It should be emphasised that only about one in 50 brothers will be diagnosed with testicular cancer and thus the absolute risk to brothers is still quite small.

Having established a strong familial association, the question arises of the extent to which this is a result of genetic susceptibility. The marked racial differences in the incidence of testicular cancer (Forman, 1989), the lack of change in incidence in migrant groups (Muir *et al.*, 1987) and the high risk of testicular cancer associated with certain congenital disorders of sexual differentiation (Verp & Simpson, 1987; Savage & Lowe, 1990) all lend support to the possibility of a genetic component.

There have been several HLA phenotyping studies of unselected patients with testicular cancer (De Wolf *et al.*, 1979; Majsky, 1979; Pollack *et al.*, 1982; Oliver *et al.*, 1986*a*; Dieckmann & Keyserlingk, 1988; Kratzick *et al.*, 1989) and, although not entirely consistent in their results, they have hinted at an association between cancer risk and specific antigens brought about by genetic disequilibrium. This association would indicate a linkage between a candidate predisposition gene and the HLA complex on chromosome 6. Anecdotal reports (Pollack *et al.*, 1982; Tollerud *et al.*, 1985; Kandel *et al.*, 1988; Hayakawa *et al.*, 1986; Oliver *et al.*, 1986*b*; Natsume *et al.*, 1989; Dieckmann & Keyserlingk, 1989) of a higher than expected level of HLA haplotype sharing between affected familial cases lend support to this hypothesis.

The sib-pair analysis (Haseman & Elston, 1972; Thomson & Bodmer, 1976) is a powerful tool to investigate genetic linkage in this type of situation where extensive pedigrees are not available. The method has been used successfully in studies of a similar size to this one to look at the association between HLA and both Hodgkin's disease (Hors *et al.*, 1984) and nasopharyngeal carcinoma (Lu *et al.*, 1990).

Our analysis, based on 21 sib-pairs, shows that the level of association with testicular cancer was no higher than that expected by random segregation. This was so despite the assumption of maximum possible sharing in families where there was ambiguity about the number of haplotypes shared. Combining the 21 pairs with the ten reported previously in the literature resulted in four, 21 and six pairs sharing 0, 1 and 2 haplotypes compared with expected numbers of 7.75, 15.5 and 7.75 for which the chi-square (4.16) is non-significant. The lack of association in our 21 pairs remained after sub-dividing sib-pairs into different histological categories, i.e., sib-pairs with only seminoma or only other germ cell tumour histologies. There was also no difference in either histological type or age at disease onset between those sib-pairs that shared two haplotypes and those that shared one or none. It is, therefore, unlikely that there is a major gene associated with testicular cancer predisposition within or linked to the major histocompatability complex. However as class II typing has not been performed we cannot formally exclude an association with class II loci that is not detectable by linkage to class I loci, similar to the situation in Hodgkin's disease (Bodmer *et al.*, 1989). In addition there remains the possibility that HLA type might have prognostic significance once the disease has developed (Oliver *et al.*, 1986*a*). We are currently investigating linkage with loci on other chromosomes.

The authors would like to thank, first and foremost, the patients and their relatives who agreed to be interviewed and have blood samples taken. We also thank the patients' General Practitioners and numerous hospital histopathology and medical records staff who made information available to us. The following made the study possible by providing us with preliminary information about relevant families: Dr F.M. McGurk, Dr H. Yosef, Department of Radiotherapy, Belvedere Hospital, Glasgow; Dr J.J. Mold, Department of Radiotherapy, Queen Elizabeth II Hospital, Birmingham; Dr H. Eckert, Oncology Department, Bristol Royal Infirmary, Bristol; Dr E.S. Newlands, Oncology Department, Charing Cross Hospital, London; Dr P.M. Wilkinson, Dr R. Gibb, Pharmacology Department, Christie Hospital and Holt Radium Institute, Manchester; Dr C.J. Alcock, Dr A.C. Jones, Dr A.H. Laing, Dr C.H. Paine, Department of Radiotherapy and Oncology, Churchill Hospital, Oxford; Dr H.J. Close, Dr W.G. Jones, Dr R.I. Rothwell, Regional Radiotherapy Centre, Cookridge Hospital, Leeds; Dr P.F. Wale, Department of Radiotherapy, Derbyshire Royal Infirmary, Derby; Dr M.A. Richards, Department of Medical Oncology, Guy's Hospital, London; Dr A.W. Jackson, Department of Radiotherapy, Norfolk and Norwich Hospital, Norfolk; Dr P.H. Cole, Radiotherapy Department, Northampton General Hospital, Northampton; Dr C.J. Tyrrell, Radiotherapy Department, Plymouth General Hospital, Plymouth; Dr J.S. Bunting, Dr F.A.L. Kircher, Department of Radiotherapy, Royal Berkshire Hospital, Reading; The late Dr H.J.G. Bloom, Dr D. Dearnley, Professor A. Horwich, The late Professor T.J. McElwain, Professor M.J. Peckham, Ms G. Jay, Royal Marsden Hospital, Sutton, Surrey; Dr A. Folkes, Department of Radiotherapy, St Luke's Hospital, Guildford; Dr G.M. Mead, Oncology Department, Southampton General Hospital, Southampton; Dr C.A.E. Coulter, Department of Radiotherapy, St Mary's Hospital, London; Dr M.A. Cornbleet, Dr G.C.W. Howard, Department of Oncology, Western General Hospital, Edinburgh; Professor S.B. Kaye, Department of Oncology, Western Infirmary, Glasgow; Dr N.J. Hodson, Dr G. Deutsch, Department of Radiotherapy, Royal Sussex County Hospital, Brighton; Dr A. Drury, Dr K. Newton, Dr R.H. Phillips, Department of Radiotherapy, Westminster Hospital, London; Dr G. Marason, Myrtle Beach Airforce Base, South Carolina, USA; AVM R.T.B. Jones; RAF Halton; Dr R. Cartwright, Dr L. Williams, ICRF Genetic Epidemiology Laboratory, Leeds.

The following interviewers helped question families and obtain blood samples: Audrey Ardern-Jones, Alison Allen, Susan Collyer, Elizabeth Hilton, Margery Thorne, Sally Reid. Dr T. Bishop, Dr G. Draper and Mrs K. Bunch provided helpful statistical advice. Mr P. Krusa and Mrs L. Kennedy provided laboratory support and Dr R. Hawkins and Dr N. Spurr carried out the DNA tests for monozygocity. Miss H. Powell typed the manuscript. The Institute of Cancer Research receives support from the Cancer Research Campaign and the Medical Research Council.

### References

AMERICAN CANCER SOCIETY (1990). *Cancer facts and figures – 1990*. American Cancer Society: Atlanta.

ANDERSON, D.E. (1975). Familial susceptibility. In Fraumeni, J.F. (ed.) *Persons at high risk of cancer*. Academic Press: New York. pp. 39–54.

BODMER, W.F. & BODMER, J.G. (1979). Cytofluorochromasia for HLA A, B, C and DR typing. In Ray, J.G. (ed.) *NIAID Manual of tissue typing techniques*. Natural Institute of Health: Bethesda. pp. 46–54.

BODMER, J.G., TONKS, S., OZA, A.M., LISTER, T.A. & BODMER, W.F. (1989). HLA-DP based resistance to Hodgkin's disease. *Lancet*, **i**, 1455.

BODMER, W.F. (1982). Cancer genetics. *Cancer Surv.*, **1**, 1.

BRESLOW, N.E. & DAY, N.E. (1980). *Statistical Methods in Cancer Research*. Volume 1 – The analysis of case-control studies. International Agency for Research on Cancer: Lyon.

CANCER RESEARCH CAMPAIGN (1990). *Facts on cancer – fact sheet 1.2, Incidence – UK – 1985*. Cancer Research Campaign: London.

CHILVERS, C.E.D. & PIKE, M.C. (1989). Epidemiology of undescended testis. In Oliver, R.T.D., Blandy, J.P. & Hope-Stone, H.F. (eds) *Urological and Genital Cancer*. Blackwell Scientific Publications: Oxford. pp. 306–321.

DE WOLF, W.C., LANGE, P.H., EINARSON, M.E. & YUNIS, E.J. (1979). HLA and testicular cancer. *Nature*, **277**, 216.

DIECKMANN, K.-P., BOECKMANN, W., BROSIG, W., JONAS, D. & BAUER, H.-W. (1986). Bilateral testicular germ cell tumors. Report of nine cases and review of the literature. *Cancer*, **57**, 1254.

DIECKMANN, K.-P., BECKER, T., JONAS, D. & BAUER, H.W. (1987). Inheritance and testicular cancer. Arguments based on a report of 3 cases and a review of the literature. *Oncology*, **44**, 367.

DIECKMANN, K.-P. & VON KEYSERLINGK, H.J. (1988). HLA association of testicular seminoma. *Klin. Wochenschr.*, **66**, 337.

DIECKMANN, K.-P. & VON KEYSERLINGK, H.J. (1989). HLA typing in familial testicular cancer. *Eur. Urol.*, **16**, 361.

EASTON, D. & PETO, J. (1990). The contribution of inherited predisposition to cancer incidence. *Cancer Surv.*, **9**, 395.

FORMAN, D. (1989). Epidemiology of testis cancer. In Oliver, R.T.D., Blandy, J.P. & Hope-Stone, H.F. (eds). *Urological and Genital Cancer*. Blackwell Scientific Press: Oxford, pp. 289–305.

GEDDE-DAHL, T., HANNISDAL, E., KLEPP, O.H. & 5 others (1985). Testicular neoplasms occurring in four brothers. A search for a genetic predisposition. *Cancer*, **55**, 2005.

GOSS, P.E. & BULBUL, M.A. (1990). Familial testicular cancer in five members of a cancer-prone kindred. *Cancer*, **66**, 2044.

HASEMAN, J.K. & ELSTON, R.C. (1972). The investigation of linkage between a quantitative trait and a marker locus. *Behav. Genet.*, **2**, 3.

HAYAKAWA, M., MUKAI, K., NAGAKURA, K. & HATA, M. (1986). A case of simultaneous bilateral germ cell tumors arising from cryptorchid testes. *J. Urol.*, **136**, 470.

HORS, J., BONAITI-PERRIE, C., D'AGACY, M.F. & 5 others (1984). Hodgkin's Disease. In Alberts, E. & Meyers, W. (eds). *Histocompatibility Testing*. Springer-Verlag: Berlin. pp. 411–414.

KANDEL, L.B., HEISE, E.R., WOODRUFF, R.D., HARRISON, L.H., MCCULLOUGH, D.L. & DYER, R.B. (1988). Testicular germ cell tumours in HLA-genotype non-twin brothers. *Urology*, **32**, 315.

KNUDSON, A.G. (1986). Genetics of human cancer. *Ann. Rev. Genet.*, **20**, 231.

KRATZIK, C., AIGINGER, P., KUZMITS, R. & 4 others (1989). HLA-antigen distribution in seminoma, HCG-positive seminoma and non-seminomatous tumours of the testis. *Urol. Res.*, **17**, 377.

LU, S.-J., DAY, N.E., DEGOS, L. & 8 others (1990). Linkage of a nasopharyngeal carcinoma susceptibility locus to the HLA region. *Nature*, **346**, 470.

MAJSKY, A., ABRAHAMOVA, J., KORINKOVA, P. & BEK, V. (1979). HLA system and testicular germinative tumours. *Oncology*, **36**, 228.

MOSTOFI, F.K. & SOBIN, L.H. (1977). *International histological typing of testes tumours* (No. 16). World Health Organisation: Geneva.

MUIR, C., WATERHOUSE, J., MACK, T., POWELL, J. & WHELAN, S. (1987). *Cancer Incidence in Five Continents* – Volume V. International Agency for Research on Cancer: Lyon.

NATSUME, O., OZONO, S., TSUMATANI, K. & 4 others (1989). Testicular tumors occurring in non-twin brothers: a case report. *Jpn. J. Clin. Oncol.*, **19**, 72.

OLIVER, R.T.D. (1990). Atrophy, hormones, genes and viruses in aetiology of germ cell tumours. *Cancer Surv.*, **9**, 263.

OLIVER, R.T.D., STEPHENSON, C.A., PARKINSON, M.C. & 4 others (1986*a*). Germ cell tumours of the testicle as a model of MHC influence on human malignancy. *Lancet*, **i**, 1506.

OLIVER, R.T.D., STEPHENSON, C.A., PARKINSON, M.C. & 4 others (1986*b*). Germ cell tumours of the testicle. A model of MHC influence in human malignancy. *J. Immunogenet.*, **13**, 85.

OFFICE OF POPULATION CENSUSES AND SURVEYS (1990). *Cancer statistics: registrations, England and Wales, 1985*. Series MBI no 18. Her Majesty's Stationery Office: London.

PATEL, S.R., KVOLS, L.K. & RICHARDSON, R.L. (1990). Familial testicular cancer: report of six cases and review of the literature. *Mayo Clin. Proc.*, **65**, 804.

PETO, R., PIKE, M.C., ARMITAGE, P., BRESLOW, N.E. & 6 others (1976). Design and analysis of randomised clinical trials requiring prolonged observation of each patients. I. Introduction and design. *Br. J. Cancer*, **34**, 585.

POLLACK, M.S., VUGRIN, D., HENNESSY, W., HERR, H.W., DUPONT, B. & WHITMORE, W.F. (1982). HLA antigens in patients with germ cell cancers of the testis. *Cancer Res.*, **42**, 2470.

PUGH, R.C.B. (1976). (ed.) *Pathology of the Testis*. Blackwell: Oxford.

RAGHAVAN, D., JELIHOVSKY, T. & FOX, R.M. (1980). Father-son testicular malignancy. Does genetic anticipation occur? *Cancer*, **45**, 1005.

SAVAGE, M.O. & LOWE, D.G. (1990). Gonadal neoplasia and abnormal sexual differentiation. *Clin. Endocrinol.*, **32**, 519.

SWERDLOW, A.J., HUTTLY, S.R.A. & SMITH, P.G. (1987). Testicular cancer and antecdent disease. *Br. J. Cancer*, **55**, 97.

SWERDLOW, A.J. (1986). Cancer registration in England and Wales: some aspects relevant to interpretation of the data. *J. R. Statist. Soc. A*, **149**, 146.

THOMSON, G. & BODMER, W.F. (1976). The genetic analysis of HLA and disease associations. In Dausset, J. & Svejgaard, A. (eds). *HLA and Disease*. Munksgaard: Copenhagen. pp. 84–93.

TOLLERUD, D.J., BLATTNER, W.A., FRASER, M.C. & 9 others (1985). Familial testicular cancer and urogenital developmental anomalies. *Cancer*, **55**, 1849.

VERP, M.S. & SIMPSON, J.L. (1987). Abnormal sexual differentiation and neoplasia. *Cancer Genet. Cytogenet.*, **25**, 191.

WEISSBACH, L. & WIDMANN, T. (1986). Familial tumor of the testis. *Eur. Urol.*, **12**, 104.